# *“I was treated like a human being”*: factors influencing women's preference for traditional birth attendants in Nigeria

**DOI:** 10.3389/fgwh.2026.1720050

**Published:** 2026-05-18

**Authors:** Abena Asefuaba Yalley, Khadijah Sanusi Gumbi

**Affiliations:** 1 Department of Politics and Public Administration, University of Konstanz, Konstanz, Germany; 2 Zukunftskolleg, University of Konstanz, Konstanz, Germany; 3 Department of Political Science, Bayero University, Kano, Nigeria

**Keywords:** childbirth, health-seeking behavior, maternal health, obstetric violence, traditional birth attendants, women

## Abstract

**Introduction:**

Nigeria's maternal mortality ratio is the highest on the African continent, and the most severe in the world, accounting for more than a quarter (28%) of global maternal deaths. A key factor in the high maternal deaths is the low utilization of facility-based services for childbirth. Despite national and global efforts to promote skilled birth attendance, about half of Nigerian women still resort to Traditional Birth Attendants (TBAs) for childbirth. Understanding the barriers that hamper the use of Skilled Birth Attendants is crucial to reducing maternal mortality and morbidity in Nigeria. The aim of this study is to explore the factors that influence women's preference for Traditional Birth Attendants over formal health facilities in Nigeria.

**Methodology:**

This is a qualitative study conducted in Kano and Oyo States in Nigeria. Specifically, Focus Group Discussions were conducted with women who have utilized TBAs for childbirth, community leaders, Civil Society Organizations (CSOs) and stakeholders. The research participants were purposively selected using the snowball sampling technique. The Dedoose software for analysis was utilized to generate codes from the data, and the analysis was thematically conducted according to Braun and Clarke's framework.

**Results:**

The analysis highlights the intersection of socio-cultural, economic, and structural factors that sustain the prominence of TBAs, despite awareness of the potential health risks. Six main themes that significantly shaped women's choice for Traditional Birth Attendants were: (1) economic factors, (2) sociocultural and religious beliefs and practices, (3) mistreatment and abuse in health facilities, (4) compassionate care from TBAs, (5) lack of education and awareness, (6) accessibility and health system issues. While all these factors determined women's preference for Traditional Birth Attendants, the most profound is the abuse and dehumanizing treatment women receive from healthcare practitioners. This is contrary to the compassionate, women-centered, and respectful care that TBAs provide, making TBAs a preferred choice.

**Discussion:**

The study concludes that effective maternal health policies and practices in Nigeria must integrate context-specific approaches that address cultural sensitivities, strengthen compassionate care and foster community-level trust in skilled birth attendants.

## 1 Introduction

Pregnancy and delivery problems cause the deaths of approximately 197 women per every 100,000 live births worldwide (1). Most of these maternal deaths occur in developing countries, especially in sub-Saharan Africa, which bears two-thirds of the burden (1, 2). Nigeria alone accounts for more than a quarter (28%) of the global maternal mortality rate, ranking the highest in Africa for total maternal deaths (2, 3). According to the Integrated African Health Observatory (2), the country's maternal mortality ratio, estimated at 917 per 100,000 live births in 2017, increased by approximately 14% to 1,047 deaths in 2020. Although maternal mortality is a huge problem, the greater proportion of the deaths is preventable through quality maternal care provided by Skilled Birth Attendants (SBAs). Poor referral systems, postpartum hemorrhage, pregnancy-induced hypertension, inadequate infrastructure and equipment, and delays in seeking healthcare have all been linked to high maternal mortality rates in several countries (4). In Nigeria, pre-eclampsia, postpartum hemorrhage, protracted or obstructed labor, infections such as maternal sepsis, and excessive bleeding before the onset of labor (antepartum hemorrhage) are among the most common causes of maternal mortality (3). Scientific evidence has demonstrated that skilled birth attendance can reduce more than 70% of maternal deaths (5–7), making SBAs the center of global action on maternal mortality. The World Health Organization (WHO) defines a Skilled Birth Attendant as a licensed health professional such as a doctor, nurse, or midwife, trained to identify, manage, and refer women and newborn babies with complications, as well as handle uncomplicated pregnancies, deliveries, and the immediate postpartum period (8). Hence, to achieve the global Sustainable Development Goal of a maternal mortality rate of 70 per 100,000 live births by 2030, the use of SBAs during childbirth is crucial.

In Nigeria, the use of SBAs is generally low. Available statistics indicate that only 43% of Nigerian women utilize the services of SBAs for childbirth (9). The skilled birth attendance coverage also differs across the care continuum. Specifically, 67% of pregnant women utilize ante-natal care, while only 38% women receive postnatal care from a qualified birth attendant within two days after delivery (9). The use of SBAs also varies by State, with Northern States having less coverage than Southern States. A population-based survey comparing SBA use in three Northern States (Katsina, Yobe, and Zamfara) demonstrated a high percentage of home births with TBAs in Katsina (82.3%), as well as rates of 95% and 87.2% in Zamfara and Yobe respectively (10). Traditional Birth Attendants are individuals who have learned the skill of conducting deliveries informally, usually through apprenticeship with older TBAs (11). While TBAs generally provide significant support, comfort, familiarity, and a sense of cultural sensitivity for women during childbirth, there are significant health risks linked to their usage. The fluid nature upon which delivery skills are acquired makes them incapable of early detection and management of birth complications, which could put the lives of women at risk. In particular, the lack of medical training needed to recognize and handle life-threatening complications during childbirth, and the lack of equipment to address these issues is major setback (12, 13). This often causes a delay in transferring mothers with complications who need immediate care to healthcare facilities, increasing the risk of maternal or neonatal deaths (12, 13). In Nigeria, studies reveal the unprecedented popularity of TBAs and the vast home births managed by unqualified and unskilled individuals significantly contribute to the high maternal and neonatal mortality rates (14). These home-based deliveries often occur without access to emergency obstetric care, skilled supervision, or proper sanitation, which are essential for reducing maternal deaths. Despite the risks associated with the utilization of TBAs, it remains a profound choice for many women. A study conducted in Eastern Nigeria established that, of the 93% of rural women who registered for prenatal care, a significant 49% still chose to give birth at home with the support of TBAs (15). Similarly, another study indicated approximately 30% of women in the Southwestern Nigeria had their childbirths attended by TBA (16).

Understanding the factors for the low utilization of SBAs has been a focal point of maternal health research in Nigeria. Studies reveal that the widespread use of TBAs is linked to various socio-economic and accessibility factors. These include poverty, limited access to healthcare facilities, and low education levels (14, 17, 18). TBAs are generally seen as more affordable than formal healthcare providers, making them the preferred choice for women from poorer economic backgrounds. In many rural areas, the costs of transportation to a hospital, consultation fees, and medical procedures can be extremely high. As a result, women often choose TBAs, who are locally available and charge little or nothing for their services (19–21). Ebuehi and Akintujoye (18) established in their studies that a woman's occupation, especially being a housewife or unemployed, strongly predicts her likelihood of using TBAs. This connection is based on the idea that these women have fewer personal financial resources and may lack the power to make healthcare decisions. Additionally, economic constraints greatly influence women's health-seeking behaviors in Nigeria (22, 23). To mitigate these challenges, the Nigerian government has made some significant efforts to increase accessibility and reduce the cost of obstetric care. The government has implemented a number of interventions, such as the Subsidy Re-investment and Empowerment Programme (SURE-P) and Midwives Services Scheme (MSS) to address the workforce shortage. Also, the Basic Health Services Scheme (BHSS) and Primary Health Care Under One Roof were initiated to deal with accessibility challenges. To mitigate the financial burden of childbirth, the government has also implemented the free Maternal and Child Health policy and the Maternal Mortality Reduction Innovation Initiative, which eliminates user fees for pregnant women. Yet, Nigeria's maternal mortality continues to rise, and more women persistently use TBAs.

Despite recent efforts to improve maternal health outcomes in Nigeria, a significant gap remains in understanding the persistent reliance on TBAs in spite of the availability of formal medical facilities. While existing literature often focuses on physical distance and poverty, there is a lack of qualitative evidence exploring the intersection of cultural norms, perceived mistreatment, and the “human dignity” trade-offs women navigate during childbirth. This study aims to explore these nuanced drivers from the perspectives of mothers, community leaders, and healthcare stakeholders, centering women's voices. Consequently, this study seeks to address the following specific research questions: (1) what socio-cultural and structural factors influence the preference for TBAs over formal healthcare facilities among women in Nigeria? (2) How do perceptions of “human dignity,” mistreatment, and informal costs shape the decision-making process for maternal healthcare utilization? The results of the study are critical to enabling stakeholders to design appropriate policies and interventions to increase the use of SBAs in Nigeria in order to reduce the high maternal mortality.

## 2 Materials and methods

### 2.1 Study design and setting

The qualitative research method was utilized to explore the factors influencing women's preference for TBAs in Nigeria. The qualitative approach is particularly appropriate for exploring a specific phenomenon thoroughly to acquire detailed information concerning personal experiences and perspectives (24), offering a critical understanding of the underpinning factors for the utilization of TBAs. The Study was conducted in Kano and Oyo States, located in two distinct geopolitical zones in Nigeria (Northwest and Southwest). These States were selected in order to obtain diverse research participants with regards to their socio-economic, cultural, tribal, and religious backgrounds. Kano State, located in the northern part of Nigeria has deeply rooted cultural traditions shaped mainly by the Hausa-Fulani heritage and Islamic Religion. Kano is noted for strict social and gender norms mainly rooted in the Islamic religion. Conversely, Oyo State in the Southern Nigeria is dominated by the Yoruba tribe with a blend of Christian, Islamic and Traditional Religion and liberal social and gender norms. According to the National Population Council (25), Kano and Oyo States have approximately 24 million inhabitants, and the rate of skilled birth attendance is about 51% (16), suggesting that about half of the women give birth through TBAs. Empirical data were collected in both rural and urban settlements. Specifically, the research was conducted in Ibadan North Local Government Area, Ibadan South-West Local Government Area, Kano Metropolis, and Danzabuwa in the Bichi Local Government.

### 2.2 Study population and sampling

The researchers utilized a Focus Group Discussion (FGD) guide to explore the views and experiences of mothers who have utilized TBAs for childbirth, community leaders, Civil Society Organization (CSO) members and stakeholders in the selected States. FGDs allow in-depth, interactive inquiry that reveals the underlying meanings, diverse perspectives, and contextual factors shaping human attitudes and behaviors (26). Altogether, five FGDs were conducted with a total of 36 participants. Each discussion consisted of a minimum of five and a maximum of ten participants. All the participants were women (except group five with one male). The mothers were mainly between ages 18 and 45 and mostly petty traders. In Kano, participants were all Hausas while the greater majority of participants in Ibadan were Yorubas. In terms of religion, all the participants in Kano were Muslims while Oyo had a blend of Muslims and Christians. The participant distribution in each group is shown in Supplementary Table S1.

The purposive sampling method was used to ensure that only participants who met the inclusion criteria were selected, while the snowball sampling was adopted to identify hard-to-reach participants. The inclusion criteria involved women who have utilized the services of TBAs for childbirth and are living in the study communities, women community leaders who are involved in community leadership roles, members of Civil Society Organizations who work on maternal and reproductive health, and stakeholders in public health institutions. The study excluded women who had no experience with TBAs or lived outside the research setting, women who occupied no leadership positions in the communities, civil society organizations whose work were not related to reproductive health and stakeholders who worked in private health facilities. In the context of this study, the views and experiences of mothers were crucial in understanding the factors that influence their choice of TBAs for delivery. The discussion with community leaders, CSOs and stakeholders provided a rich insight into the existing socio-cultural norms and other institutional factors. The selection of the sample size and method was based on saturation. The data collection ceased when new discussions did not generate any new insight or information, making the sample size sufficient for examining the drivers of TBA utilization.

### 2.3 Data collection procedure

The FGDs were conducted between August 2024 and January 2025. The principal investigator recruited and trained three research assistants (who had at least a bachelor's degree with experience in qualitative research) to assist with the data collection. The training centered on the protocol, sampling procedure, ethical issues and qualitative research techniques. The recruitment of mothers and community leaders was made in the communities, while the stakeholders were recruited from CSOs and healthcare institutions. The researchers first made contact with community leaders, explaining the rationale for the study. Community leaders who accepted to participate recommended other leaders and mothers. Some of the mothers were also recruited in their homes by researchers based on the recommendation of other mothers. The researchers approached the mothers and CSO members and those who were willing to participate were invited to the discussion. The recruitment of stakeholders was made in the health facilities and ministries, after an initial meeting with administrators for approval. Each FGD was conducted by two researchers (a moderator and a rapporteur who took notes). To ensure safety, privacy and open discussion, the discussion was conducted in a secure and safe room in a rented facility within the community. To avoid dominant voices and ensure equal participation, the groups were fairly homogenous and each participant was given the opportunity to respond to each of the discussion questions. The FGD guide captured the demographic data of participants, experiences with TBAs, and the factors influencing the utilization of TBAs. The discussions were conducted in Hausa, English and Yoruba languages, with each discussion lasting for about two hours. All the discussions were audio recorded with consent from all participants.

### 2.4 Research rigor

Rigor of the research findings was established through reflexivity, debriefing, audit trail and transferability. As African women with childbirth experiences in Nigeria, the researchers were conscious of how their experiences, personality and perceptions could influence the research. To mitigate potential biases, the researchers adopted multiple participants (mothers, community leaders, CSOs and stakeholder) to ensure objectivity. The researchers recognized that participants might perceive them as “government officials”. To mitigate this, the researchers established their position as academic researchers prior to the discussion and dressed in a manner consistent with local norms to build rapport and reduce the perceived power gap. The purposive sampling ensured that only research participants who possessed the required experiences and knowledge were recruited. Also, debriefing, through constant peer examinations were conducted to discuss the research process, to ensure that the research protocol was strictly adhered. To maintain audit trail and transferability, the research protocol was adhered to and the research procedure, process and methods are thoroughly reported to ensure possible replication of this research in the future.

### 2.5 Data analysis

The reflexive thematic data analysis approach was utilized to analyze the data. Specifically, the researchers adopted Braun and Clarke's (27) framework for thematic analysis- identification of patterns and emerging themes in the data to answer the research questions. First, the oral discussions were first translated and transcribed into English and back-checked for consistencies and errors. The transcripts were then thoroughly read, and reread for familiarity. Thereafter, the data were exported into Dedoose software for data analysis. One researcher (AAY) and a secondary coder coded the data on Dedoose software. Codes and sub-codes were generated through the inductive approach to identify common and emerging themes. For this paper, only codes that focused on factors influencing the utilization of TBAs were extracted and analyzed. Major themes and sub-themes were derived from the codes. To ensure transparency and mitigate confirmation bias, the transition from raw data to final themes followed a clear “code-to-theme” progression. Specifically, open coding was used to identify recurring phrases followed by thematic generation through the clustering of sub-themes and the construction of major codes. The coders met to discuss the results to ensure consensus and validate the findings (which was strictly based on the raw data). Data saturation was assessed by monitoring the emergence of new codes during the final FGDs in Kano and Oyo. By the fourth group, no novel conceptual insights were appearing, suggesting that the primary drivers of healthcare-seeking behavior in this context had been captured.

### 2.6 Ethical considerations

This study was approved by the Ethics Committee of the University of Konstanz, Germany (IRB 43/2024). In Nigeria, ethical approval was granted by the Oyo State Ministry of Health Research Ethics Committee (NHREC/OYOSHRIEC/10/11/22) and Kano State Ministry of Health (NHREC/17/03/2018). In addition, written and verbal consent was obtained from all participants before the discussion.

## 3 Results

**Figure 1:**
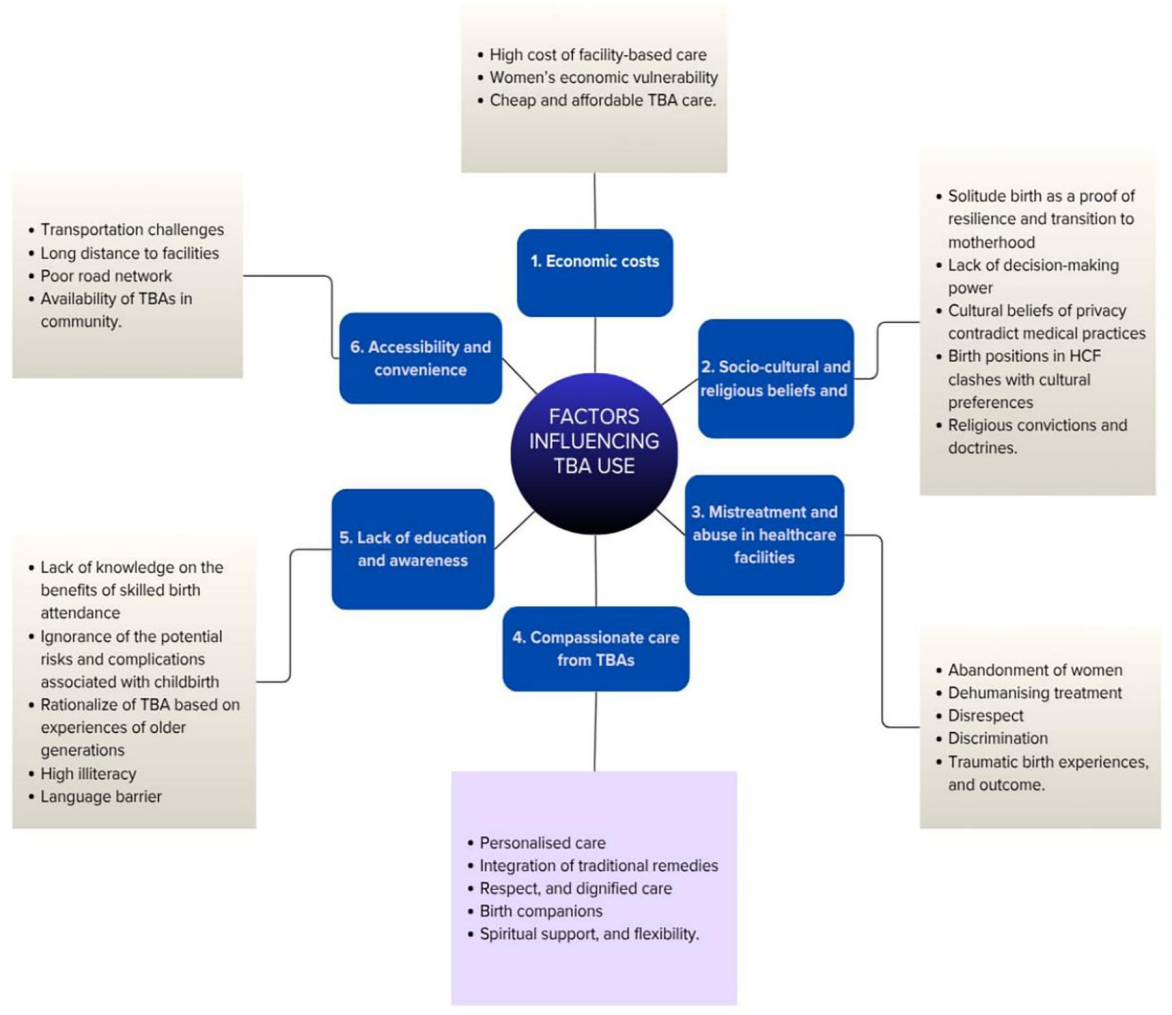
Thematic framework of factors influencing TBA utilization.

This study explored the factors influencing women's preference for TBAs for childbirth in Nigeria. Figure 1 shows the themes emerging from the study analysis.

### 3.1 Economic factors

The financial cost of healthcare was identified by the participants as a significant determinant in women's decision to utilize TBAs. Healthcare in Nigeria is generally expensive. In particular, access to quality healthcare is even more expensive. Although the government provides some subsidies for maternal health in public health facilities, the poor quality of care and the subsequent requests for diverse materials for birthing make facility-based care unattractive for many women. The evidence suggests that, despite official costs, healthcare facilities often levy additional charges or request provisions such as kerosene, coconut oil, and other miscellaneous supplies, contributing to the overall financial burden and making childbirth in health facilities unaffordable for many families. According to one of the participants:

They always say delivery is free at Murtala hospital, but when you go there, they will ask you to buy things like kerosene, coconut oil, and other items. By the time you add everything, it is no longer free, and many families cannot afford it. (Participant, Mother)

Another woman reiterated:

The government says free delivery, but in practice, you must spend money. They will tell you to bring many items, and poor families struggle with that. That is why most women prefer the TBAs. (Participant, Community Leader)

Women's economic disempowerment further reinforces this preference as cost becomes the primary factor in their healthcare decisions regarding childbirth, often overshadowing the potential medical benefits of facility delivery. Furthermore, the issue of financial constraints is inextricably linked to broader issues of poverty and a lack of economic empowerment among women. This economic dependence often curtails women's autonomy in making critical healthcare decisions, including the choice of birth attendants and delivery location. In many instances, husbands or other influential family members exercise control over household finances and consequently dictate where a woman will give birth, often prioritizing cost over other factors such as the availability of comprehensive medical care and emergency obstetric services.

Unfortunately, their men do not want to pay money. Instead, they will go to the farm with the money and will not assist the women, which is the biggest problem. He will be like, “Why would I take my money and use it to take you to the hospital? After all, if you were to boil some herbs and take them, you would give birth. After all, one of the wives did the same and gave birth.” That is the number one issue. (Participant, Civil Society Organization)

When I told my husband I wanted to go to the hospital, he said the money was too much and he would rather use it for farming. He told me, “Other women gave birth at home without spending anything, why can’t you do the same?” So, I had no choice but to deliver at home. (Participant, Mother)

In contrast, TBAs are widely regarded as a more economically viable option. The cost is typically lower than healthcare institutions and is often subject to negotiations, providing a degree of financial flexibility that aligns with the socioeconomic realities of many pregnant women and their families. This affordability makes TBAs an accessible alternative, particularly in communities where financial resources are limited.

The TBAs are affordable. Before my wife gave birth to the twins, the hospital said I should pay over a hundred thousand naira. When we used the local women, they just asked for a few items, like palm oil, Kerosene and a few items. And everything went smoothly. (Participant, Stakeholder)

Some TBAs return your things to you if you do not finish them but hospitals demand for too much and they are called public. Was it the government that told them to be acting like that? (Mother)

From the foregoing, it is evident that financial limitations heavily favor TBAs over Skilled Birth Attendants due to the high and unpredictable costs, including informal charges. As demonstrated, some stakeholders even use TBAs due to financial constraints.

### 3.2 Sociocultural and religious beliefs and practices

Cultural norms, particularly in Northern Nigeria, mandate women to undergo childbirth at home, either in solitude or with the support of families or relatives. This practice is sometimes viewed as a significant cultural rite, marking a woman's transition into motherhood and serving as a powerful display of her physical and emotional resilience. These deeply ingrained traditions can be resistant to change, even in the face of potential medical benefits offered by modern healthcare facilities. The following extracts from the discussions reiterate the cultural dimensions of home births.

Sometimes, when a woman is pregnant, they make her give birth alone in the room. The woman finishes everything alone and then comes out with the baby. Here in Kano, we do this thing. Many people say it is in their family house that they give birth, so you [woman] have to give birth in your room, you alone. It is a cultural thing. Once you are done, you bring out the baby, even if it is your first child. They will just show you the procedure. You will be given a mortar or a chair and be told to hold it with all your might because that is your only support. That is the way to prove that you are a woman. (Participant, Civil Society Organization)

When I was in labor, I begged my family to take me to the hospital because the pain was too much, but they refused. In our community, it is seen as a serious abomination for a woman to deliver in the hospital. No matter the problem, they say you must stay at home and give birth the way our mothers and grandmothers did. Even when I was weak and afraid, they told me it would bring shame to the family if I went to the hospital. So, I was forced to remain at home with the traditional birth attendant, because that is what our culture demands, even if the woman's life is at risk. (Participant, Mother)

Again, in many communities, the husband and older female relatives, particularly mothers-in-law, wield considerable influence over younger women's healthcare decisions during pregnancy and childbirth. These senior family members often exert pressure on expectant mothers to adhere strictly to traditional birthing practices, viewing hospital births with skepticism. This pressure can stem from a desire to preserve cultural heritage, a lack of awareness about the risks associated with home births, or negative perceptions of modern medical interventions.

Some of the participants added that;

There is the influence of mothers-in-law. They tell the young women, “I myself, all my kids, I gave birth more than fourteen times, and I never went to the hospital.” It shows that you, the young pregnant woman is not strong or brave enough. (Participant, Stakeholder)

Also, some of the men have this perception that their mothers did not visit the hospital, and as such, their wives will not go to the hospital. They also think we have all these TBAs that have been practicing this thing for a long time and many women have given birth in their hands, so there is no need to spend much [in the hospital]. (Participant, Civil Society Organization)

Furthermore, specific beliefs and concerns surrounding hospital environments can act as significant deterrents for women seeking facility-based obstetric care. The Islamic religion holds strong reservations about women being cared for by male healthcare providers during such a personal and vulnerable experience. Concerns about privacy, modesty, cultural and religious appropriateness influence many families to choose TBAs or home births. Some participants sharing their experiences revealed;

For us, it is not acceptable for another man, especially a young doctor, to see the nakedness of the wives during childbirth. Even if she is sick or in labor, our culture and religion teach us that her body should be protected. That is why many men will not agree to take their wives to the hospital, because they know that in most cases, the doctors there are men. Instead, they prefer to send their wives to the traditional birth attendant, where only women will attend to them and their privacy will be respected. (Participant, Community Leader)

When I told my husband I wanted to deliver in the hospital, he refused immediately. He said, ‘Do you want another man to touch you in such a condition?’ In our community, people will laugh at a man whose wife is treated by male doctors, saying he has no shame. So even if the hospital has better care, many husbands will not allow their wives to go, because they fear the loss of modesty and respect. That is why we end up with the TBAs, who are always women. (Participant, Mother)

Religious beliefs also exert a notable influence on childbirth practices in many communities. Certain religious groups have their distinct birthing rituals, traditions, or guidelines that favor home births or the involvement of specific religious figures. In some instances, religious teachings may discourage the use of hospitals or modern medical interventions during childbirth, emphasizing faith-based approaches to pregnancy and delivery. These deeply held religious convictions can significantly shape a woman's choices regarding where and how she gives birth. Personal and religious beliefs also play a role, with some women preferring TBAs or adhering to church-based birthing practices that may discourage institutional births.

And some also prefer churches; there are churches that have their own group of women who take deliveries. Some churches have their dos and don'ts. For example, there's a church that my auntie attended, and she gave birth to her second child at home because they don't go to the hospital. They don't. So, she was bleeding after she had the child, bleeding for almost a week and she died the day of the naming ceremony. So, because of religious reasons. (Participant, Mother)

I have three children. I gave birth to the boy at home. I'm a Christian and I already registered at the hospital. When I was seven months pregnant, I just heard [a voice] that day that I went to antenatal, “do not come to this place again”. Unknown to me, people around me heard the same thing. That is God. It is the best delivery experience I have had. That's the best I've had. (Participant, Mother)

### 3.3 Mistreatment and abuse in health facilities

The experiences of mistreatment and abuse by healthcare professionals came strongly as the most prominent factor that discourages women from utilizing healthcare facilities for childbirth. Women frequently encounter harassment, dehumanizing treatment, disrespect, and even neglect from healthcare professionals, creating a hostile environment within healthcare facilities. This mistreatment erodes trust and deters women from seeking necessary medical attention. The following accounts of dehumanizing treatments reinforce the devastating nature of obstetric violence:

A woman in labor was taken to the labor room, and when the midwives checked her, they said she was not due, but she insisted she was about to give birth. They now instructed her to go out and wait. She went out and sat on the floor. She was wearing a hijab, and underneath that hijab, she gave birth. She informed the health workers that she had given birth, and everyone there was shocked. The caregiver now came, checked her and cut the umbilical cord. After that, she instructed the woman to move aside and clean up. Clean up the space! How do you expect someone who just gave birth and was treated like this to come back to the hospital again? (Participant, Community Leader)

I have heard people saying that they went to the hospital when they were due and even fully dilated, but were turned back home. One person said the healthcare worker told her, “You don’t even know that you are not ready to give birth right now.” So, she was sent out. Due to that, they will inform others, and others will inform others. That also contributes to it. It discourages others from coming to the hospital. (Participant, Civil Society Organization)

Furthermore, discriminatory practices based on a woman's financial status or outward appearance are reportedly prevalent, leading to disparities in the quality of care received. Women perceived as wealthier or better-dressed often receive preferential treatment compared to those from lower socioeconomic backgrounds. A participant revealed that:

At times, the kind of harassment they [women] receive from the medical personnel also discourages them from going to the hospital. Most especially, our people from the core rural areas. You know, capitalism has penetrated the system. If you are not financially well-off, well-dressed, you will not receive the proper attention that you deserve. This particular issue discourages most women from rural areas. (Participant, Civil Society Organization)

The women also narrated personal experiences of abandonment by healthcare professionals when they requested help. In many instances, women who went to health facilities were abandoned to deliver without any assistance from healthcare professionals.

When my labor started, my family rushed me to the hospital. But when we arrived, the place was already overcrowded. They told me there was no bed space, so I had to lie down on the floor in the corridor, even though I was in severe pain. I kept crying for someone to attend to me, but no one came quickly. I delivered my baby there on the floor, without proper attention. That day, I told myself I would never return to the hospital again; it was too humiliating and painful. (Participant, Mother)

I thought I would get the best care, but my experience was different. I was made to wait for many hours, even though I was already bleeding and weak. They kept saying the doctors were busy, and when they finally came, they only looked at me for a few minutes and left. I felt abandoned, like my life did not matter. Later, other women in the ward told me that this is common. Since then, I prefer to go to the TBAs, because at least they stay by your side until you deliver. (Participant, Mother)

I took my Auntie to the hospital when she was in labor, and one of the nurses was like, “You are not yet dilated, you should have stayed at home.” They neglected her. She was on the chair when her water broke, and the doctor who came to attend to her ended up being splashed in the face as the baby slipped out. When she returned, she said nothing would ever bring her back to the hospital because if she could give birth on a chair, she would stay in her room, on her bed, and give birth in her comfort zone. (Participant, Mother)

Tragically, some women have endured traumatic experiences within hospital settings, such as the preventable loss of a baby due to perceived negligence. These devastating events can create lasting emotional scars and a profound distrust in the healthcare system, making women hesitant to return for future medical needs, regardless of the urgency. These negative experiences collectively contribute to a climate of fear and uncertainty, hindering women's access to essential healthcare services.

A woman told me her sad experience with how she was treated by healthcare workers. She went to the hospital when she was in labor, but the health workers insisted that she was not due for labor and sent her back home. Unfortunately, while they were going, she gave birth in the car and the baby died and she herself lost consciousness. When she regained consciousness, she realized she had VVF (Vesicovaginal Fistula), she had that disease. You know, that disease that she has, honestly, affects her life because it was her first child and she was in her first year of her marriage, and it really affected her marriage. She took an oath that she will never give birth in the hospital. Now she has two kids. She was telling me, “You see, these kids, I gave birth to them in my own room”. She said it was her mother who came and helped her and now the children are alive. She was telling me the story. She has even influenced her friends not to go to the hospital. That is what is happening. (Participant, Mother)

Conversely, a significant factor driving women towards TBAs is the lack of compassion and mistreatment of women in healthcare facilities in Nigeria. This mistreatment overshadows the medical advantages offered by health facilities, leading women to seek care from TBAs where they feel more understood and respected.

### 3.4 Compassionate care from TBAs

Unlike the SBAs, the TBAs often provide more compassionate and personalized care. They foster a sense of welcome and support for women during pregnancy and childbirth. This welcoming environment can extend beyond mere physical assistance to encompass emotional support, understanding cultural nuances, and providing practical help within the familiar context of the woman's home or community. The personalized care offered by TBAs creates a strong bond of trust, making women feel more comfortable and secure during such a vulnerable time. Women who have utilized these traditional women narrated positive childbirth experiences.

When my labor started at night, I went straight to the TBA in our community. She received me immediately, without asking me to wait. She spoke to me gently in our language and reassured me like a mother. I felt heard, I was not rushed, and she stayed by my side throughout the process. That closeness and kindness made me strong, unlike in the hospital, where you are often left alone. (Participant, Mother)

The woman took her time with me. She was patient, checking on me often, and she never shouted at me. She even allowed my husband to sit nearby, which gave me courage. I felt I was treated like a human being. (Participant, Mother)

The narratives from the women also revealed that the TBAs integrate traditional remedies and practices into their care. These practices are deeply rooted in the local culture and are often trusted by women due to familiarity passed down through generations. The use of familiar herbs, rituals, and birthing positions can provide psychological comfort and align with the woman's belief system. Because the birth attendants are natives who live in the communities, the language barriers are negated and cultural beliefs about childbirth are inculcated in the care process. The desire for a compassionate and culturally sensitive birth experience often outweighs the perceived risks associated with traditional birth practices in the minds of these women.

The TBA understands our traditions and treats us with respect. She allowed my mother and aunt to be present and even prayed with us before the delivery. I felt safe because she is one of us; she knows our language and beliefs. With her, I never felt ignored or humiliated, the way some women say they feel in the hospital. That is why many of us prefer to deliver with TBAs, despite what people say about risks. (Participant, Mother)

The woman who took my delivery respected our cultural ways of giving birth. She understood the herbs we use for strength and allowed me to pray in my own way. That made me feel at peace and cared for, unlike the hospital, where everything feels strange and rushed. (Participant, Mother)

The inculcation of spiritual freedom into care is another element that attracts women to TBAs. Most African women are spiritual, and this sense of spiritual flexibility offered in local birthing homes fulfils women's spiritual needs. For many women, birthing is not only physical and medical, but also a spiritual phenomenon.

At the birth center, the women prayed with me before delivery. That gave me confidence, and I believed God was with me. Their kindness and encouragement made my labor easier. (Participant, Mother)

The women treated me with respect and explained everything they were doing. I felt safe and spiritually supported. It was not just about giving birth, but about being cared for in body and soul. (Participant, Mother)

### 3.5 Lack of education and awareness

A lack of education and awareness about the risks of home births and the benefits of facility-based care contributes to the preference for TBAs. Many women lack sufficient knowledge regarding the potential risks and complications that can arise during childbirth, and they may not fully appreciate the critical role that skilled birth attendants play in ensuring a safe delivery for both the mother and child. This lack of awareness can lead to delayed decision-making in seeking professional medical help when complications occur, potentially resulting in adverse outcomes. During the discussion, participants disclosed that:

They don't have the knowledge about the importance of going to the hospital, and that discourages them from going to the hospital. They will just say, “It is nothing; I can do it. I have given birth several times.” They don't know the types of risks associated with giving birth, so they prefer to stay at home and give birth. (Participant, Stakeholder)

Many of the women here do not have formal education, so they don't understand the dangers of childbirth. They believe that because they have delivered three, four, or even more children at home, nothing bad will happen. Some will say, “This is natural, women have been giving birth at home since the time of our grandmothers.” They are not aware of complications like prolonged labor, bleeding, or infection. Because of this lack of knowledge, they see no reason to go to the hospital and instead prefer the TBAs who tell them what they want to hear. (Participant, Stakeholder)

For me, giving birth is something natural; I have done it many times at home without problems. When the nurses say I should go to the hospital, I just tell them, “Why should I waste money when God has helped me before?” I feel it is better to stay with the TBAs I am used to. (Participant, Mother)

Furthermore, a prevailing cultural belief exists in some communities where women rationalize their choice of home births based on the experiences of previous generations in their families. They may think that because their mothers and grandmothers successfully gave birth at home without medical intervention, they can do the same. This pervasive perspective often overlooks the advancements in obstetric care and the potential for unforeseen emergencies that require immediate medical attention, which are better managed in a healthcare facility with trained professionals. One of the respondents recounted that;

In our family, all the women before me: my mother, my grandmother, and even my elder sisters gave birth at home with the TBAs. So, when it was my turn, everyone expected me to do the same. They said, ‘This is how our women have always done it, and nothing happened to them.’ Because of that, I felt there was no need to go to the hospital. It is difficult to break away from what the older women in the family who see it as the normal way. (Participant, Mother)

Another participant disclosed:

When you look around, most of the women in this community still use the TBAs. They will tell you stories of how many children they delivered safely without stepping into a hospital. So as a young woman, you feel encouraged to follow them because you don't want people to say you are different. If other women are doing it and surviving, you believe you can also do it. That is why the TBAs continue to have many clients. (Participant, Community Leader)

Low levels of literacy among women present a significant barrier to accessing and understanding crucial healthcare information related to pregnancy, childbirth, and postnatal care. This lack of literacy can impede their ability to comprehend health education materials, follow medical instructions, or effectively communicate their health concerns to healthcare providers. Moreover, navigating the complexities of the healthcare system, including understanding referral processes and accessing available resources, becomes considerably more challenging for women with limited literacy skills, further increasing their vulnerability during pregnancy and childbirth. During the discussion, a participant emphasized:

Lack of education! The fact that in the hospital, English will be spoken to them. That line of communication is a problem. Also, the fact that they will be told to “go there or here” [referral system]. They get confused, and at the end of the day, they feel threatened or somehow harassed. So, even if they have the money, the fact that they need to go to the hospital and be spoken to in a different language than the one they understand, they decide to stay in their comfort zone. And that comfort zone is their homes, and calling the traditional women with experience in that environment. (Participant, Stakeholder)

### 3.6 Accessibility and health system issues

Long distances to healthcare facilities, compounded by a lack of reliable and affordable transportation options, present a significant barrier for women seeking essential medical services, especially during critical emergencies such as childbirth. This geographical and logistical challenge can lead to delays in receiving timely care, potentially resulting in adverse health outcomes for both mothers and their babies.

One big problem is the distance to the hospital. From our village, it can take three or more hours to reach the nearest one, and the road is very bad. Sometimes there is no transport at night, and if a woman goes into labor suddenly, it becomes almost impossible to take her there. That is why many families give up and just use the TBAs in the community. (Participant, Community Leader)

Some people often go into labor during the night. If the husband is unavailable and there is no one to help her to the hospital and there is a TBA nearby, she might opt for that for convenience sake. For instance, I always go into labor at midnight or at dawn.

(Participant, Mother)

In contrast, TBAs often reside within the communities they serve, making them geographically accessible to women, even during nighttime hours or in urgent situations where traveling to a distant hospital would be impossible. Their presence within the community fosters a sense of trust and availability, further encouraging women to seek their assistance when needed.

One of the reasons we go to the TBAs is that they are always within reach. Their houses are inside our communities, so you don't have to travel far. Even if labor starts suddenly in the night, you can just send someone to call her and she will come immediately. Unlike the hospital, where you must find transport and sometimes wait in a long queue. The TBA is there for you at any hour. That makes us feel safe, because we know help is always close by. (Participant, Mother)

## 4 Discussion

The purpose of this study was to explore the factors that influence women's choice of TBAs over Skilled Birth Attendants in Nigeria. The data indicate that the preference for TBAs is not a simple choice but a complex issue driven by a confluence of factors. The inclination towards TBAs in Nigeria is a multifaceted phenomenon rooted in a complex interplay of socioeconomic, cultural, educational, experiential, geographical, and gendered dynamics. Typically, the economic implications of skilled birth attendance and mistreatment of women in health facilities intersect with sociocultural norms to create a conducive atmosphere facilitating a strong preference for TBAs. Financial constraints present a significant barrier for many expectant mothers, as the costs associated with utilizing health facilities, including consultation fees, medication expenses, and potential transportation costs, can be prohibitive in communities grappling with poverty. Recent statistics indicate that more than 60% of the poor people in Nigeria are women, with about 75.9% of rural women being multi-dimensionally poor due to biased gender norms (28, 29). This economic reality often makes the services offered by TBAs, which are generally more affordable and sometimes provided through barter or in-kind payments, a more realistic option. Our findings corroborate with quantitative studies in Nigeria, which found that women with low income were less likely to use conventional health facilities for childbirth (30). Another study by Ogbo et al. (16) also found that women with employment have a lower odd of utilizing TBAs. As demonstrated in our study, women's financial autonomy plays a key role in determining their utilization of TBAs. These findings call for critical policies that ensure completely free quality healthcare services for pregnant women. Although the cost of maternity care is subsidized in Nigeria, it is not comprehensive. Moreover, the findings point to the need for women's financial empowerment as complete reliance on men compromises women's health.

One of the key findings of our study is how gender norms and the position of women in society are closely linked to TBA utilization. Although previous studies in Nigeria report poverty, limited access to healthcare facilities, low education levels and deep-seated cultural beliefs about childbirth as barriers to facility-based birthing (15, 19, 23), the gendered barriers were not captured. Women wield less power in their families and are therefore unable to make decisions regarding their health and choice of birthplace. The decisions are often made by the husbands or mothers-in-law, concealing women's epistemic voice. Ranked 139 out of 153 countries in the 2021 Global Gender Gap Index, Nigeria is among the lowest-performing countries in the world in terms of promoting women's rights and ensuring gender equality (31). The strong patriarchal culture silences women's voices, choices, and autonomy in reproductive health. Hence, the decision-making power rests mainly with men, to the disadvantage of women. Globally, gender remains a key determinant of health outcomes, determining the use of contraceptives and health-seeking behaviors (32). In the Nigerian context, gendered power dynamics a key factor in women's inability to utilize skilled birth attendants, which is key to the prevention of maternal mortality. Undoubtedly, dealing with the challenge of unskilled birth attendance demands a radical change in gender norms and inequalities.

Overall, the prominent factor influencing women's preference for TBAs is mistreatment and abuse during childbirth in healthcare facilities. The mistreatment experienced at health facilities deters women from seeking professional care. Women reported experiences of abandonment, dehumanizing treatment, disrespect, humiliation, and lack of privacy. These experiences and the related traumatic impacts, particularly on maternal and neonatal morbidity, create a sense of distrust and dissatisfaction, pushing women towards the compassionate and personalized care offered by TBAs. The quest for respectful maternity care is therefore the key factor to women's preference for TBAs. The findings of our research are consistent with previous studies, which have found mistreatment and abuse of women during childbirth to be highly prevalent in facility-based care in many parts of Africa. Yalley et al.'s study (33) involving over 2000 women in Ghana established that almost two in every three women who give birth in health facilities experience abuse and mistreatment. In Nigeria, Okafor (34) found that up to 98% of women who gave birth in healthcare facilities were subjected to abusive and violent treatment during childbirth. Women have reported a high incidence of physical violence (36%), which includes being restrained or tied down during labor, sutured episiotomies without anesthesia, beatings, slapping, or pinching, and being sexually abused by health workers (35). Although these mistreatments have a great impact on women's physical and mental health (35), our study found that it is a prominent reason for women's utilization of TBAs. This is consistent with Alemu et al.'s study (36) in Ethiopia, where abuse and violence during childbirth was the strongest factor associated with fear of institutional deliveries. Similarly, Hatamleh et al.'s study in Jordan established that the quest for humanized treatment during childbirth was a prominent reason for elective caesarian sections among women in Jordan (37). In Nigeria, TBAs often establish close relationships with the women they serve, offering continuous emotional support, personalized attention, and respectful maternity care, contrasting with experiences at health facilities. Hence, respectful maternity care plays a significant role in positive birth outcomes. Specifically, it increases “satisfaction, enhances facility-based deliveries, reduces anxiety and postpartum trauma, and promotes better clinical outcomes” (38:169). This compassionate and humanized care provided by TBAs is a significant draw.

Furthermore, the results of the study showed that deeply entrenched sociocultural beliefs and traditional practices exert a strong influence on birthing choices. Among the Islamic community, strict doctrines on privacy also attract women to TBAs as the healthcare facilities compromise their privacy. Moreover, health system issues such as unfamiliar language, complicated referral systems, poor road network and long waiting hours make the environment alien. In many Nigerian communities, childbirth is viewed as a natural and communal event, traditionally managed by experienced women within the community, the TBAs. These women are often respected figures, deeply integrated into the social fabric, and their practices are interwoven with cultural rituals and beliefs surrounding pregnancy and delivery. This familiarity and cultural resonance can create a sense of trust and comfort that modern health facilities, perceived as impersonal or foreign, may struggle to replicate. Also beliefs in supernatural guidance, providence, support, and healing influenced women to choose local birthing homes for childbirth. Similar research findings were reported in other parts of Nigeria. A study in the Cross River State of Nigeria found that TBAs integrate traditional rituals, such as herbal treatments and communal prayers, which resonate with cultural beliefs about spiritual protection during childbirth (39). Also, in Akwa Ibom State in Nigeria, Udoma et al. (40) found that women perceive TBAs as culturally aligned, offering personalized care that hospitals often lack. These reinforce reliance on TBAs, who adeptly blend spiritual and traditional elements to address women's fears of complications or spiritual attacks. Hence, it is important to critically review and modify birthing protocols in health facilities, allowing harmless practices that resonate with cultural and religious values, such as the presence of family members to support women, birth positions of choice, and spiritual support. As solitude home births were also considered a rite of passage and emblem of motherhood, particularly in Northern Nigeria, there is also the need for more education on the health impacts of harmful traditional practices on women.

### 4.1 Conclusion and recommendations

This study explored the factors influencing women's preference for TBAs for childbirth. The study established economic factors, accessibility and health system challenges, mistreatment and abuse of women during childbirth in health facilities, lack of education, sociocultural and religious beliefs as key factors. Overall, the mistreatment and abuse of women in healthcare facilities was the most prominent reason discouraging women from utilizing facility-based services for childbirth. On the contrary, TBAs offered respectful, compassionate and personalized care which attracted women. Financial barriers, facilitated by women's economic disempowerment and loss of autonomy, play a significant role in the choice of care. This made the affordability of TBAs more attractive. Sociocultural norms were also key influencing factors. Also, the culturally and religiously sensitive care provided by TBAs fostered trust and comfort for expectant mothers. Again, the limited awareness of the potential risks associated with unskilled birth attendance and the lack of understanding of the benefits of facility-based childbirth led women to rely on TBAs. The study also found that challenges of accessibility and other systemic issues in the health facilities pose a significant barrier.

The findings of this study underscore the need for a multi-pronged approach to encourage greater utilization of health facilities for childbirth. This approach should address financial barriers through initiatives such as health insurance that make maternal healthcare services highly affordable, engaging with community leaders and TBAs to bridge cultural gaps and promote health education. Also, there is the need for heathcare institutions to improve the accessibility and quality of care in health facilities, and foster respectful and patient-centered care which builds trust in the healthcare system. A proposed model is a unilateral collaboration where TBAs are trained to support maternal care while working in tandem with formal systems—an approach that aligns medical safety with cultural sensitivity. Also, it is important to invest in community health education, training healthcare workers on cultural sensitivity and respectful maternity care. More crucial is education and promotion of women's rights and abolishing gender norms that subjugate and oppress women's reproductive autonomy. Particularly, the empowerment of women, both culturally, financially and politically, is critical.

### 4.2 Strengths and limitations

This study delves into the multifaceted reasons behind the prevailing choice of TBAs over formal healthcare facilities for childbirth among women in Nigeria. The qualitative approach provided an in-depth understanding, uncovering a nuanced web of interconnected factors spanning economic constraints, deeply ingrained sociocultural norms, varying levels of education and health literacy, and systemic challenges within the healthcare system itself. The geographical contexts and population provided comprehensive data from diverse cultural, tribal and religious backgrounds. However, the utilization of snowball sampling in the recruitment of some participants posed the risk of gatekeeper bias and the participation of socially homogenous group. Also the qualitative nature of the study limits the generalization of the results. Future studies could explore quantitative studies on TBA preferences across multiple States in Nigeria as well as Respectful Maternity Care Models in homebirths that could be integrated into facility-based birthing.

## Data Availability

The raw data supporting the conclusions of this article will be made available by the authors, without undue reservation.
