# Supplementary material for: “I was treated like a human being”: factors influencing women's preference for traditional birth attendants in Nigeria
Source: Front Glob Womens Health. 2026 May 18;7:1720050. doi: 10.3389/fgwh.2026.1720050 (PMC13223008; doi:10.3389/fgwh.2026.1720050)
Supplement: Supplementary file 1 [file Table1.docx]

| **Variables** | **FDG 1** | **FGD 2** | **FGD 3** | **FGD 4** | **FGD 5** |
| --- | --- | --- | --- | --- | --- |
| **Profile** | Mothers | Stakeholders, Community Leaders, Members of CSO | Mothers | Mothers, Members of CSOs, Community Leaders | Members of CSOs,  Stakeholders, Community Leaders |
| **Location/State** | Urban/Rural  (Kano State) | Urban/Rural  (Kano State) | Rural/Urban  (Oyo State) | Urban/Semi-Urban  Oyo State | Urban/Semi-Urban  Oyo State |
| **No. of Participants** | 8 | 10 | 7 | 5 | 6 |
| **Age Range** | 18-38 years | 18 – 45+ years | 37-45+ | 25-45+ years | 25-45+ |
| **Gender** | Female | Female | All Female | All Female | 4 Female/ 1 Male |
| **Ethnicity** | Hausa | Hausa | Yoruba | Yoruba | Yoruba/Edo |
| **Religion** | Islam | Islam | Christians and Muslims | Christians/Muslims | Christians |
| **Language** | Hausa | Hausa | Yoruba | English | English |
| **Occupation** | Housewives, Petty traders, Artisans | Healthcare practitioners, legal practitioners, Local Leaders/NGO workers | Petty traders | Unemployed, Students, Journalist, Nutritionist, Teacher | Healthcare practitioners, NGO Workers, Journalists, |
| **Duration** | 60 Minutes | 75 Minutes | 65 Minutes | 120 Minutes | 120 Minutes |

**Participant Distribution Map**
